# Monitoring Endothelial and Tissue Responses to Cobalt Ferrite Nanoparticles and Hybrid Hydrogels

**DOI:** 10.1371/journal.pone.0168727

**Published:** 2016-12-30

**Authors:** Federica Finetti, Erika Terzuoli, Sandra Donnini, Marianna Uva, Marina Ziche, Lucia Morbidelli

**Affiliations:** 1 Dept. Life Sciences, University of Siena, Siena, Italy; 2 Dept. Biotechnology, Chemistry and Pharmacy, University of Siena, Siena, Italy; Helsingin Yliopisto, FINLAND

## Abstract

Iron oxide nanoparticles (NPs) have been proposed for many biomedical applications as *in vivo* imaging and drug delivery in cancer treatment, but their toxicity is an ongoing concern. When NPs are intravenously administered, the endothelium represents the first barrier to tissue diffusion/penetration. However, there is little information about the biological effects of NPs on endothelial cells. In this work we showed that cobalt-ferrite (CoFe_2_O_4_) NPs affect endothelial cell integrity by increasing permeability, oxidative stress, inflammatory profile and by inducing cytoskeletal modifications. To overcome these problems, NPs have be loaded into biocompatible gels to form nanocomposite hybrid material (polysaccharide hydrogels containing magnetic NPs) that can be further conjugated with anticancer drugs to allow their release close to the target. The organic part of hybrid biomaterials is a carboxymethylcellulose (CMC) polymer, while the inorganic part consists of CoFe_2_O_4_ NPs coated with (3-aminopropyl)trimethoxysilane. The biological activity of these hybrid hydrogels was evaluated *in vitro* and *in vivo*. Our findings showed that hybrid hydrogels, instead of NPs alone, were not toxic on endothelial, stromal and epithelial cells, safe and biodegradable *in vivo*. In conclusion, biohydrogels with paramagnetic NPs as cross-linkers can be further exploited for antitumor drug loading and delivery systems.

## Introduction

Magnetic iron oxide nanoparticles (NPs) have been widely investigated for many years. Due to their magnetic, electronic and optical properties, they are good candidates for future use in biomedical practice. Several applications have been proposed, as diagnostic tests, *in vivo* imaging, targeted drug delivery and tissue regeneration [1, 2]. In particular, superparamagnetic iron oxide NPs (SPIONs), as cobalt ferrite NPs, have been developed for magnetic resonance imaging (MRI), magnetic intracellular/interstitial hyperthermia and magnetic drug targeting, thus they are proposed both as therapeutic and theranostic agents [3] and at the present big efforts are conducted to develop clinically relevant drug delivery systems.

Delivery devices have to be inert and non toxic for various cell types and should not induce any adverse reaction, such as immune system activation or stromal reactivity, when implanted *in vivo*. Indeed, a major limitation of drug delivery systems is their toxicity on cells and tissues into which they are designed to be injected or implanted, including endothelium.

The vascular endothelium plays an important role in maintaining cardio-vascular homeostasis, and represents the first barrier for drugs and drug delivery systems to tissue diffusion and penetration [4]. The integrity of microvascular endothelial cells after iron oxide NPs intravenous administration has been only partially examined [5, 6].

Our attention focused on newly developed polysaccharide-based hydrogels containing magnetic NPs, recently proposed as local devices for antitumor drug release [7, 8]. Previous research led to synthesis of a magnetic hybrid polysaccharide hydrogel that could release a drug when alternate magnetic fields (AMF) were applied [9, 10, 11]. The organic part is a carboxymethylcellulose (CMC) polymer and the inorganic part consists of cobalt-ferrite (CoFe_2_O_4_) NPs coated with (3-aminopropyl)trimethoxysilane (APTMS) to obtain CoFe_2_O_4_-NH_2_ NPs. The coated system acts as cross-linker of the CMC polysaccharide chains by forming amido covalent bonds. While CMC safety and biodegradability are well established [12], the compatibility and safety of magnetic silanized NPs is controversial [13, 14].

The aim of this study was first to evaluate the viability and functions of endothelial cells when exposed to CoFe_2_O_4_ and CoFe_2_O_4_-NH2 NPs and second the *in vitro* and *in vivo* safety profile of hybrid hydrogel (CMC with and without CoFe_2_O_4_-NH_2_ NPs).

## Materials and Methods

### Materials and reagents

Aqueous dispersions of cobalt ferrite NPs were provided by Colorobbia (Florence, Italy). The size of NPs is 18,6±0.15 nm, while the size of aggregated NPs in the hydrogel is 100±40 nm. Hybrid hydrogels were prepared and characterized for their physico-chemical properties as described [7, 15, 16]. The sodium salt of CMC, average MW 700 kDa, ~0.9 carboxymethyl groups per anhydroglucose unit, was from Sigma-Aldrich (Milan, Italy). Briefly, CoFe_2_O_4_ NPs were functionalized by APTMS to obtain free amino groups on their surfaces (CoFe_2_O_4_-NH_2_) NPs. The groups could react with the carboxylic acid groups of the polysaccharide, forming amido groups. NPs in the aqueous dispersions and inside the hydrogels form aggregates with an average size around 100 nm [11]. The weight of NPs constitutes 50% of the weight of the polymer, since the crosslinking reaction that leads to NPs-CMC formation, performed with less than 50% NPs does not lead to the formation of a stable hydrogel. The magnetic properties of NPs did not vary significantly after silanization and hydrogel formation [17].

A further CMC hydrogel (used as control) was prepared using 1,3-diaminopropane as cross-linking agent, as previously reported [18]. The presence of NPs increases mechanical characteristics of the hydrogel (hydrogel without NPs presents the storage or elasticity modulus (G’) 500±50 Pa and the loss or viscosity modulus (G”) 60±30 Pa, instead the G’ of hydrogel with NPs is 3300±300 Pa and the G” is 90±20 Pa) [19].

In all the experiments performed, the control condition was represented by cells without any treatment since the biomaterials under investigation were without any organic and potentially toxic solvent.

### Cell lines

Human umbilical vein endothelial cells (HUVEC) were purchased from Lonza (Basel, Switzerland) and grown in complete endothelial growth medium (EGM-2) (Lonza), supplemented with 10% fetal bovine serum (FBS) (Hyclone, Euroclone, Milan, Italy). MDA-231 mammary tumor cells were sourced from ATCC and maintained in DMEM (4500 mg/l glucose) with 10% FBS. Mouse fibroblasts (NIH-3T3), obtained from ATCC, were maintained in DMEM with 1000 mg/l glucose (Euroclone, Milan, Italy) and supplemented with 10% bovine calf serum (BCS) (Hyclone). Cells were cultured at 37°C in 5% CO_2_. They were split 1:3 twice a week, and used until passage 10.

### Cell number

Biomaterials under investigation [CoFe_2_O_4_ NPs, CoFe_2_O_4_-NH_2_ NPs, CMC gel, CMC gel + CoFe_2_O_4_ NPs or CoFe_2_O_4_-NH_2_ NPs] were sterilized by UV exposure (20 min under hood). Cells were seeded at a density of 2.5x10^4^ on immunofluorescence coverslips located in 24 well multiplates. After 24 h, NPs or lyophilized gels were added to culture medium containing 10% serum at a dose of 0.25 or 0.5 mg/ml, respectively, allowing direct contact with the cells. After 3 days of incubation, cells were fixed with 100% methanol and stained with hematoxylin and eosin (H&E) to monitor cell morphology and number. Live and attached cells were counted randomly in 10 fields/sample.

### MTT assay

HUVEC were seeded at the density of 1500/well in 96 multiwell plates in medium with 10% serum. After adhesion, cells were treated with NPs or lyophilized gels. After 68 h, medium was removed and cells were incubated for 4 h with fresh medium in the presence of 1.2 mM MTT (3-(4,5-dimethylthiazol-2-yl)-2,5-diphenyltetrazolium bromide) (Sigma, Milan, Italy). Alive cells reduce MTT to a strongly pigmented formazan product. After solubilisation in DMSO, absorbance of the formazan was measured with a microplate reader (SpectraFluor, Tecan, San Jose, CA, USA) at 540 nm. Data are expressed as absorbance units (Abs) [20].

### Western blot

Endothelial cells were seeded at the density of 50.000/well in 24 multiwell plates in medium with 10% serum. After cell adhesion, NPs or lyophilized biomaterials were dissolved in medium and placed in direct contact with the cells. After 24 h incubation, biomaterials were gently removed and cells were lysed as described in [20]. Cell lysates were centrifuged at 10000 g for 20 min at 4°C. Following gel electrophoresis, proteins were blotted onto activated nitrocellulose membranes, incubated overnight with the anti caspase-3 antibody (1:1000) (Cell Signaling, Danvers, USA), anti p21 antibody (1:1000, Cell Signaling), anti p53 antibody (1:1000) (Santa Cruz, Heidelberg, Germany), anti inducible nitric oxide synthase (iNOS, 1:1000, Santa Cruz) or anti cyclooxygenase-2 (COX-2) antibody (1:1000) (Cayman, Ann Arbor, USA). The primary antibody was detected by incubating the membranes for 1 h with horseradish peroxidase-conjugated anti-mouse or anti-rabbit antibody (Promega, Madison, USA)) diluted 1:2500 in PBS, followed by enhanced chemiluminescence detection system (Bio-Rad, Hercules, USA). Images were digitalized with CHEMI DOC Quantity One. Results were normalized to those obtained by using an antibody anti β-actin (1:10000) (Sigma). Original blots are reported as Supplemental Information S1 Fig.

### Immunofluorescence analysis

Endothelial cells (5x10^4^ cells/well on glass cover-slips placed into 24 multiwell plates) were maintained in 10% FBS for 24 h. Then cells were treated with NPs or lyophilized gels (0.25 mg/ml and 0.5 mg/ml, respectively, 24 h) and fixed in acetone for 5 min. After blocking of unspecific bindings with 3% bovine serum albumin (BSA), cells were incubated overnight at 4°C with the primary antibody (anti β-actin, 1:70, Sigma). Samples were then incubated with a secondary antibody TRITC conjugated (Sigma) and analyzed by confocal microscopy (Zeiss LSM 700) at 60X magnification [21].

### Reactive oxygen species (ROS) measurement

ROS levels were evaluated as previously reported [22]. 1.5×10^3^ cells (HUVEC) were seeded in 96-multiwell plates and, after adherence, were treated with NPs (0.25 mg/ml, 2 h) in a medium without phenol red. DCFH2-DA (2,-7-dichlorodihydrofluorescein diacetate) (Invitrogen, Milan, Italy) was added (10 μM, 30 min) and intracellular levels of ROS were evaluated photometrically with a microplate reader (excitation/emission 495/527) (SpectraFluor, Tecan). Results are reported as relative fluorescence units (RFU) corrected for the cell number counted.

### Endothelial permeability

HUVEC were seeded at 1 X 10^5^ on gelatin-coated insert membranes (Corning, New York, USA) with 0.4 μm diameter pores, and the inserts were placed in 12 multiwell plates. After 48 h, confluent monolayers were treated with NPs (0.25 mg/ml), then 3 KDa FITC-Dextran (10 μM) was added on top of cells, allowing the fluorescent molecules to pass through the cell monolayer toward the lower compartment. The extent of permeability was determined after 30 min by measuring the fluorescence, in the medium present in the bottom of the well, in a multiplate reader (SpectraFluor, Tecan), at 485/535 nm, excitation/emission, respectively. Results are reported as relative fluorescence units (RFU) [21].

#### Safety of implanted materials in animals

The effects of hybrid hydrogels were tested on C57 black mice (7–8 weeks, 20–25 g body weight; Charles River Italia, Calco, Italy) which were kept in temperature- and humidity-controlled rooms (22°C, 50%) with lights on from 7 am to 7 pm and water and food ad libitum. All experimental procedures, conducted according to Italian law (Legislative Decree n.26, 4 March 2014), which reflects European Directive 2010/63/EU, were approved by the University of Siena ethical board and the Italian Ministry of Health. All efforts were made to minimize the number of animals used and their suffering and distress.

The biomaterials investigated *in vivo* were: CMC gel and CMC gel + CoFe_2_O_4_-NH_2_ NPs. The implants were cylinders (approx. 3 x 5 mm) sterilized by UV exposure for 20 min before implantation. The biomaterials (0.5 mg/mouse) were implanted subcutaneously in C57 black mice under anaesthesia in sterile conditions. The animals were anaesthetized (ip) with a mixture of Zoletil^®^ (0.2 mg/kg tiletamine and zolazepam, Virbac, Milan, Italy) and Xilor^®^ (0.2 mg/kg xylazine, Bio98, San Lazzaro (BO), Italy). Dorsal hair was shaved and the skin wiped with 70% ethanol. An incision (2–3 mm long) was made in the dorsal skin, the biomaterials were implanted aseptically in the subcutaneous pouch and the wound was sutured with thread. Animals were housed singly to avoid the possibility of reciprocal aggression and damage to the wounds. Post-operatively, the animals were monitored for any sign of infection at the wound site, discomfort or distress. After 7 days, they were sacrificed with CO_2_ and the subcutaneous tissue and implant were exposed and photographed. The implanted material with the skin directly above it was removed, embedded in optimum cutting temperature (OCT) compound, frozen in isopentane cooled in liquid nitrogen and stored at -80°C.

### Histo-morphological evaluation

The standard histological technique of H&E was used to examine the tissue response to the implanted material. Histology of the remnant biomaterial and surrounding skin was performed on isolated and OCT-embedded tissues. Seven μm sections were prepared with a cryostat, stained with H&E and examined by light microscope. Blind assessments of the histological sections were performed by two observers. Pictures were taken at magnifications of 20X and 63X.

### Statistical analysis

All values are expressed as mean ± SEM. Statistical analysis was performed by using one-way analysis of variance (ANOVA) and Bonferroni as post-test, or two-way ANOVA and Bonferroni as post-test where appropriate (Graph-Pad software, San Diego, CA). Differences were considered statistically significant with a p value < 0.05.

## Results

### In vitro evaluation of the effect of NPs on endothelial cells

Nanoparticles are proposed for diagnostic or therapeutic use and, upon administration, the endothelial cells are the first barrier to tissue distribution. To understand the impact of NPs on vascular integrity, we investigated their activity on endothelial cells *in vitro*. We exposed endothelial cells (HUVEC) to CoFe_2_O_4_ NPs or CoFe_2_O_4_-NH_2_ NPs, functionalized by APTMS to obtain free amino groups on their surfaces, allowing their further functionalization.

**Fig 1:**
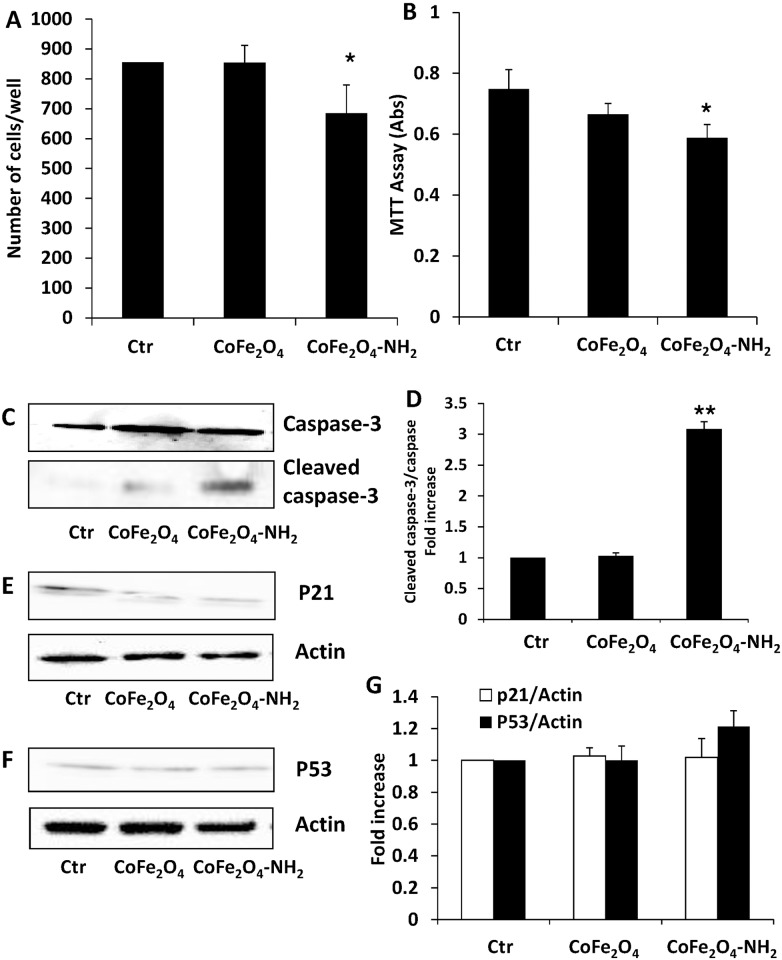
(A) Endothelial cell number after incubation for 3 days with CoFe_2_O_4_ or CoFe_2_O_4_-NH_2_ NPs. Data are means±SEM; *p<0.05 vs Ctr. (B) Endothelial cells survival was evaluated by MTT test. Cells were exposed to CoFe_2_O_4_ NPs or CoFe_2_O_4_-NH_2_ NPs for 3 days and data are expressed as absorbance at 540 nm. *p<0.05 vs Ctr. (C, E, F) Expression of markers of apoptosis and cell cycle arrest in HUVEC exposed to NPs. Cells were exposed to the biomaterials for 24 h and then lysed. Cleaved caspase-3 (C), p21 (E) and p53 (F) expression was measured by western blot. Blots are representative of 3 experiments with overlapping results. (D, G) Data in the graph represent the quantification of the protein of interest vs total caspase-3 or beta actin, and are expressed as fold increase vs Ctr. **p<0.0 vs Ctr.

Firstly, cell survival and growth were studied after culture in contact with CoFe_2_O_4_ NPs or CoFe_2_O_4_-NH_2_ NPs (0.25 mg/ml) for 3 days. As shown in (Fig 1), CoFe2O_4_-NH_2_ NPs modestly reduced the number (Fig 1A) and survival (Fig 1B) of HUVEC, whereas no significant effect was observed for CoFe_2_O_4_ NPs.

**Fig 2:**
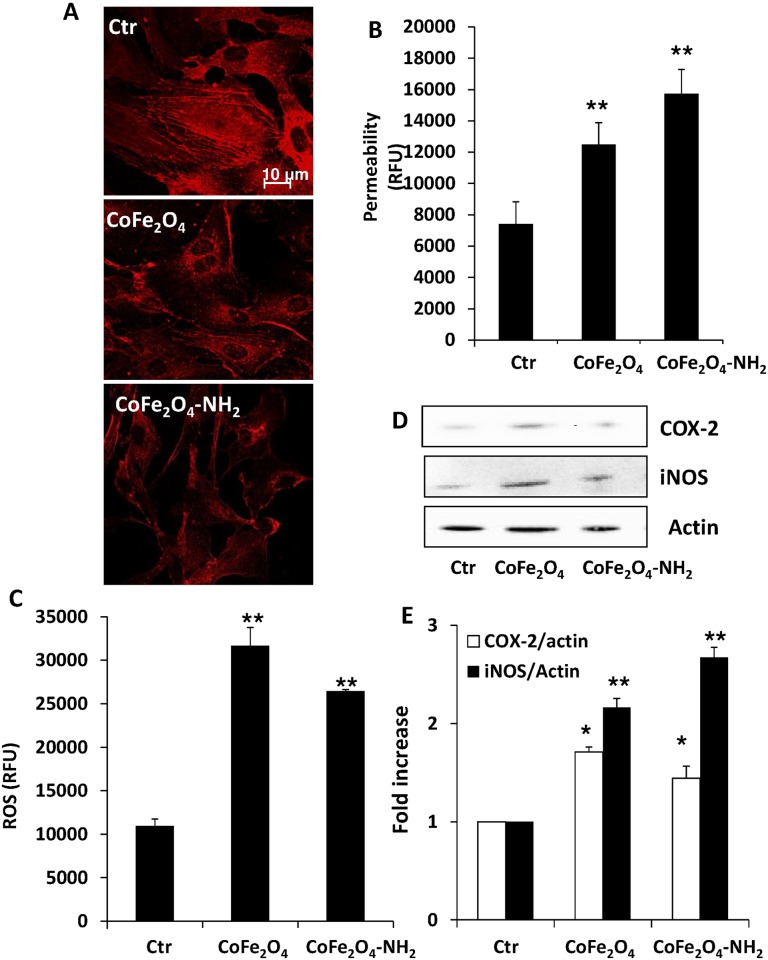
(A) Immunofluorescence analysis of actin in HUVEC treated for 24 h with CoFe_2_O_4_ NPs or CoFe_2_O_4_-NH_2_ NPs (0.25 mg/ml). (B) Permeability in HUVEC monolayers was detected as passage of FITC-dextran from upper to lower compartment of a transwell. Data are expressed as relative fluorescence units. **p<0.01 vs Ctr. (C) ROS measurement after 2 h of exposure with the different NPs. Data are expressed as relative fluorescence units. **p<0.01 vs Ctr. (D-E) Expression of COX-2 and iNOS in HUVEC exposed for 24 h to CoFe_2_O_4_ NPs or CoFe_2_O_4_-NH_2_ NPs (0.25 mg/ml) measured by western blot. Blots are representative of 3 experiments with overlapping results. (E) Data in the graph represent the quantification of the protein of interest vs beta actin and are expressed as fold increase vs Ctr. *p<0.05 vs Ctr and **p<0.0 vs Ctr.

In view of these results, to investigate the mechanisms underlying reduction in endothelial cell number and survival, we analysed some molecular pathways in cells activated after treatment with the different NPs, focusing on the pro-apoptotic signal caspase-3 and proteins involved in cell cycle regulation. In particular, we evaluated caspase-3 activation, cleaved in apoptosis, and the expression of p53, involved in regulation of cell growth by holding the cell cycle at G1/S phase. Blockade of cell proliferation promoted by p53 is also linked to increased expression of inhibitory proteins, such as p21. After treatment for 24 h with CoFe_2_O_4_ NPs or CoFe_2_O_4_-NH_2_ NPs (0.25 mg/ml), we observed caspase-3 activation (Fig 1C and 1D) promoted by CoFe_2_O_4_-NH_2_ NPs, whereas p53 and p21 protein levels resulted unchanged (Fig 1E, 1F and 1G). These data clearly indicate that CoFe_2_O_4_-NH_2_ NPs promote the activation of apoptotic pathways. The phenotype and cytoskeleton organization of HUVEC were analysed by confocal microscopy. Actin organization, after 24 h of incubation with CoFe_2_O_4_ NPs or CoFe_2_O_4_-NH_2_ NPs, appeared modified by both types of NPs (Fig 2A). While in untreated cells β-actin was organized in stress fibres sustaining/encompassing the whole cell body, in treated cells stress fibres disappeared and labelling was distributed in the peripheral actin rim, as sign of cell quiescence.

Considering the physiological barrier function of endothelium, we evaluated the properties of NPs in affecting transcellular permeability of endothelial monolayer. As shown in Fig 2B, CoFe_2_O_4_ NPs or CoFe_2_O_4_-NH_2_ induced a robust increase of permeability, that was associated with higher intracellular ROS levels (Fig 2C) and induction of pro-inflammatory enzymes (Fig 2D and 2E). Indeed, endothelial cells exposed to NPs for 24 h showed increased levels of COX-2 and iNOS (Fig 2D and 2E), indicating that microvascular integrity was affected by NPs treatment.

### In vitro evaluation of the effect of hybrid hydrogels on cell cultures

Since free NPs showed several deleterious effects on cultured endothelium, we decided to study hybrid hydrogels in which NPs (sylanized and not) have been loaded with carboxymethyl cellulose (CMC), verifying their *in vitro* and *in vivo* safety profile.

**Fig 3:**
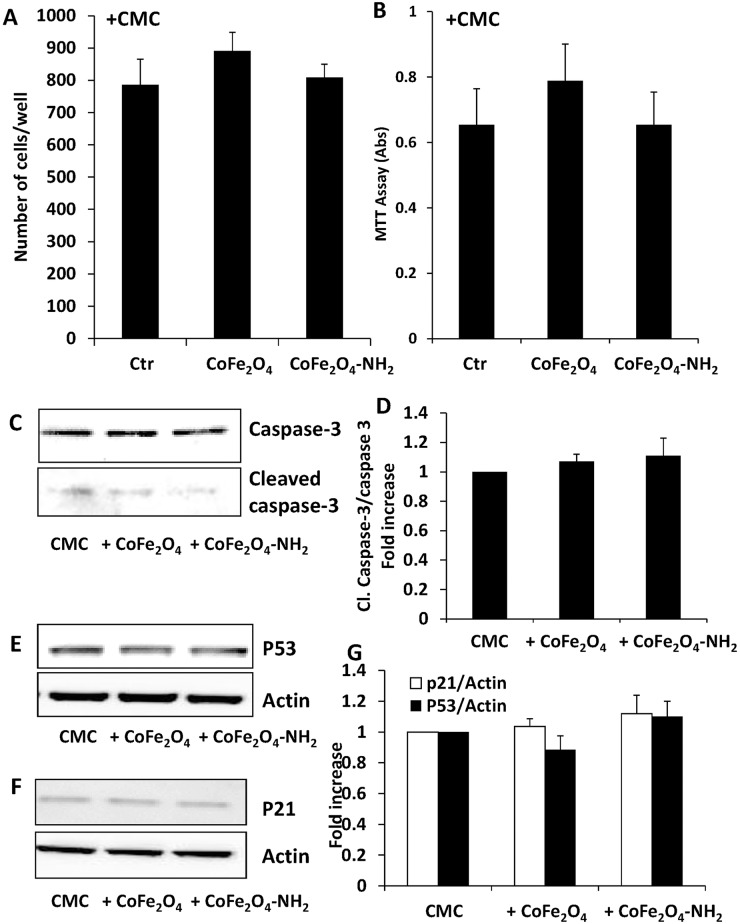
(A) Endothelial cell number after incubation for 3 days with CMC alone, CoFe_2_O_4_-CMC or CoFe_2_O_4_-NH_2_-CMC (0.5 mg/ml). Data are means±SEM. (B) ECs survival was evaluated by MTT test. Cells were exposed to CMC, CoFe_2_O_4_-CMC or CoFe_2_O_4_-NH_2_-CMC (0.5 mg/ml) for 3 days and data are expressed as absorbance at 540 nm. (C-G) Evaluation by western blot of the expression of markers of apoptosis (C), cleaved caspase-3 and cell cycle arrest (E, p53, and F, p21) in HUVEC exposed to CMC, CoFe_2_O_4_-CMC or CoFe_2_O_4_-NH_2_-CMC (0.5 mg/ml) for 24 h. Blots are representative of 3 experiments with overlapping results. (G) Data in the graph represent the quantification of the protein of interest vs total caspase-3 or beta actin, and are expressed as fold increase vs Ctr.

**Fig 4:**
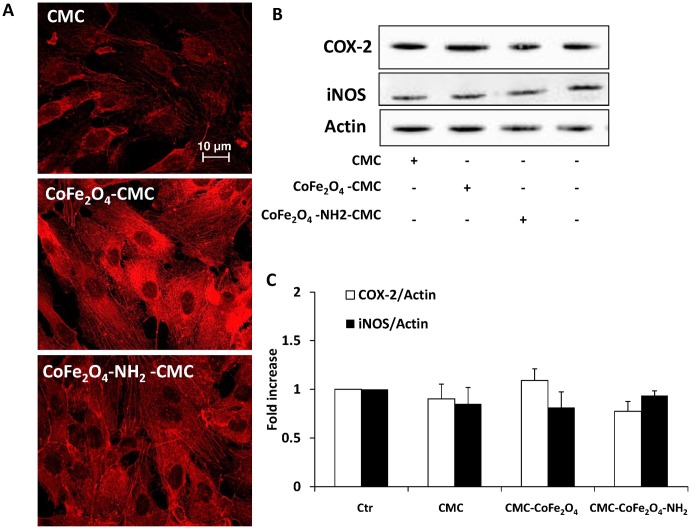
(A) Immunofluorescence analysis of actin in HUVEC treated for 24 h with CMC, CoFe_2_O_4_-CMC or CoFe_2_O_4_-NH_2_-CMC (0.5 mg/ml). (B-C) Expression of COX-2 and iNOS in HUVEC exposed for 24 h to the biomaterials measured by western blot. Blots are representative of 3 experiments with overlapping results. Data in the graph represent the quantification of the protein of interest vs beta actin and are expressed as fold increase vs Ctr.

These hybrid biomaterials were first evaluated on HUVEC proliferation and survival after 3 days of culture. In both type of experiments CMC alone did not alter either the number of cells or the % of living cells in the MTT test respect to not treated cells (survival: 853.04±15 and 785.9±59 cells/well; MTT: 100% and 87.7±9, control vs CMC alone, respectively). In addition, CoFe_2_O_4_-CMC or CoFe_2_O_4_-NH_2_-CMC (0.5 mg/ml) did not modify the number and vitality of endothelial cells (Fig 3A and 3B) respect to CMC alone and, consistently, we did not observe caspase-3 activation (Fig 3C and 3D) or modification of p53/p21 expression (Fig 3E, 3F and 3G). Furthermore, when we analysed cytoskeleton organization or inflammatory profile of HUVEC after 24 h of exposure to hybrid hydrogels, we did not detect any difference among CMC alone and CMC loaded with NPs. β-actin was organized in stress fibres in all the three samples and relevant inflammatory enzymes were not upregulated, also respect to untreated cells (Fig 4A, 4B and 4C). Since these biomaterials are proposed for local cancer treatments, a model of stromal cells (mouse NIH-3T3 fibroblasts), and a model of epithelial tumor cells (MDA 231) were also analysed. In these cell models, NPs embedded into CMC gel did not affect cell number (Table 1).

**Table 1:** Effect of NPs loaded CMC on cultured fibroblasts and breast carcinoma cells.

	CMC	CoFe_2_O_4_-CMC	CoFe_2_O_4_-NH_2_-CMC
**NIH-3T3**	100%	107±4.5%	96±2.8%
**MDA 231**	100%	99.5±3.2%	88.4±8.9%

Data are reported as percentage of cell number counted after 3 days of treatment with hybrid hydrogels; CMC alone, CoFe_2_O_4_-CMC or CoFe_2_O_4_-NH_2_-CMC (0.5 mg/ml). The number of cells in control conditions (10% serum) was: 125±18 for NIH-3T3 and 38±9 for MDA 231 (n = 3).

On the whole, these results document the safety of the hybrid hydrogels under investigation. It is noteworthy that the presence of CMC or low percentages of NPs did not *per se* exert any *in vitro* toxicity on stromal or tumor cells, indicating that these biomaterials could safely be exploited for drug delivery.

### Assessment of in vivo safety of hybrid hydrogels

Subcutaneous hybrid hydrogel implants (0.5 mg/sample) were performed in C57 black mice to evaluate the safety, inflammatory reaction and biodegradation of the matrix. Implants were performed under general anaesthesia after skin incision with a surgical scalpel in the dorsal area and wound closure with suture thread. The materials investigated were CMC gel and CoFe_2_O_4_-NH_2_-CMC (0.5 mg/ml). The animals were observed daily for wellness and behaviour, and no relevant modification in their routine activities was found.

All the biomaterials were well tolerated. No signs of infection or rejection were observed in the implant location during the 7-day period of the experiment as the implants became progressively infiltrated by fibrovascular tissue.

**Fig 5:**
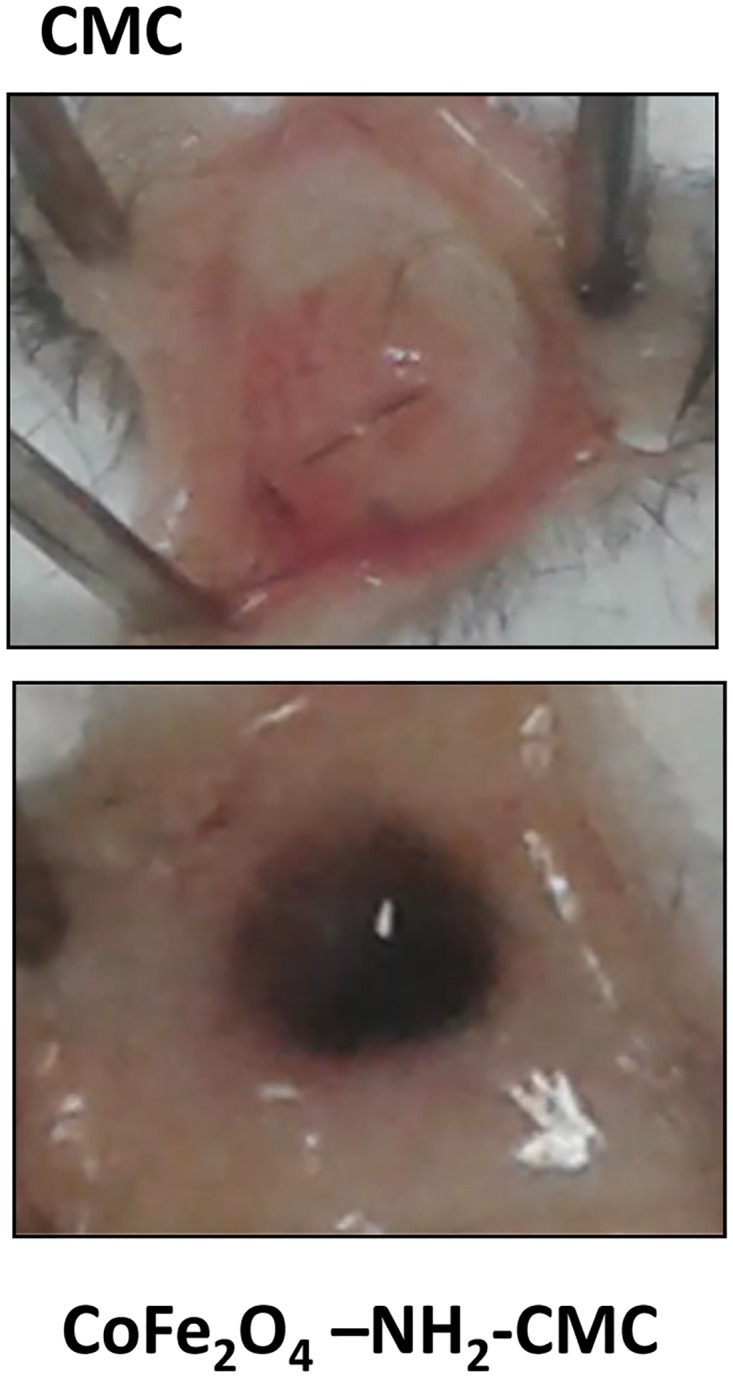
Macroscopic analysis of subcutaneous implants of hybrid hydrogels in mice. Sterilized biomaterials were implanted subcutaneously in C57 black mice under anaesthesia. Surgical implants were closed with suture thread. Animals were sacrificed 7 days after implant; the subcutaneous implant was exposed and photographed.

**Fig 6:**
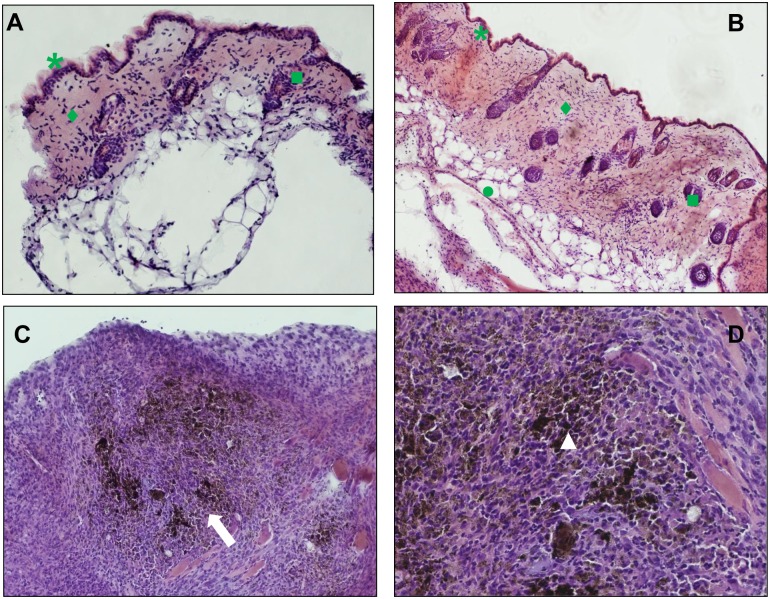
Histological analysis of skin and subcutaneous tissue around the implant. Panels a and b are representative images of dermis and epidermis near implants of CMC and CoFe_2_O_4_-NH_2_-CMC, respectively. Panels c and d show tissue reactivity to the biomaterials. The CMC scaffold is no longer visible probably due to biodegradation. Representative images at 20X (A, C) and 63X (B,D) magnification. Legend: *epidermis, ♦ dermis, ■ hair follicles. Arrows indicate inflammatory cell infiltration (neutrophils, macrophages/monocytes, lymphocytes). Nanoparticles are clearly visible in phagocytes (white arrowheads).

Seven days after surgery, the implants and surrounding skin were photographed (Fig 5), sampled and processed for histology. The skin close to all implants was normal and healthy. Non conjugated CMC gel was resorbed into subcutaneous adipose tissue, since the implant appeared faint and hollow, however it can not excluded that the residual CMC hydrogel has been dissolved during sample staining processes. No inflammatory cell infiltrate was found by microscopic observation of tissue sections (Fig 6A and 6B). While CMC implant was safe with no adverse reaction, CoFe_2_O_4_-NH_2_-CMC induced inflammatory cell infiltration, in particular neutrophils and monocytes/macrophages (Fig 6C and 6D).

These *in vivo* data document that hybrid hydrogels are biologically processed: although the CMC scaffold is degraded, the NPs remain in the subcutaneous tissue where they induce an inflammatory cell infiltrate at the site of implantation.

## Discussion

New drug delivery systems to avoid side effects of several drugs are an ongoing field of research, rising many concerns. Drug delivery systems based on NPs have significant advantages, such as: i) the ability to target a specific location in the body, avoiding the possibility of passive drug release; ii) reduction of drug concentrations at non-target sites, thus minimizing systemic toxic side effects; iii) reduction of costs, an important factor for innovative drugs [23]. Coating NPs with a biocompatible polymer, such as polysaccharides, increases circulatory half-life from minutes to hours or even days and reduces spontaneous aggregation of NPs [24, 25]. Moreover, polysaccharide hydrogels have been conceived as drug delivery devices for local treatment, with the possibility of incorporating nanomaterials or NPs [15, 16]. Recently, magnetic NPs have been proposed as an innovative method to drive new or classical drugs toward a specific organ site (i.e. in cancer therapy). Particularly, cobalt ferrite NPs are one of the most promising candidates for medical application, for their peculiar physical and mechanical properties.

In this manuscript, an innovative nanocomposite hybrid material has been engineered. This material should combine the advantages of having magnetic NPs able to be driven to and maintained nearby the target site with the presence of a polysaccharide-based matrix which renders the material injectable and well tolerated by the patient. Moreover, the presence of the NPs makes the material capable of responding to a magnetic stimulus which should induce structural changes in the materials and hence release the drugs. The material could be employed for targeted release of drugs effective against primary and secondary bone tumors. Secondary bone cancers would greatly benefit from the use of biomedical devices which could transport and release antineoplastic drugs close to the target. However, the devices could also be useful for either treating primary bone cancers that could not be surgically removed or for preventing in situ relapses of osteosarcomas after surgery. However, only few reports consider the effects of NPs, in general, and cobalt ferrite NPs, in particular, on vascular endothelium. In this work we show that CoFe_2_O_4_ NPs and CoFe_2_O_4_-NH_2_ NPs display robust endothelial toxicity by increasing endothelial permeability, oxidative stress, cytoskeleton organization and inflammatory markers, suggesting that intravenous administration of cobalt ferrite NPs alone should be avoided.

To that end, we decided to investigate the biological properties and safety of a hybrid hydrogel for local therapy, as described. The results here reported demonstrate that CMC hydrogel is not toxic and harmful for cultured cells; when implanted *in vivo*, it is safe and biodegradable. Consistently, the viability of different types of cells, including stromal, endothelial and tumor cells, is not affected by CMC CoFe_2_O_4_ NPs and CoFe_2_O_4_-NH_2_ NPs. In addition, treatment of vascular endothelium with this hybrid hydrogel does not modify endothelial viability, cytoskeleton organization and inflammatory marker expression.

Moreover, our *in vitro* findings support the subcutaneous inflammatory cell infiltrate *in vivo*. Indeed, we show that neutrophils and macrophages infiltrate subcutaneous tissue near the implants of CMC combined with NPs and internalize NPs released by CMC degradation. These data are in line with previous reports demonstrating the cytotoxicity of superparamagnetic iron oxide NPs in murine fibroblasts [13] and their accumulation in stromal cells, such as fibroblasts, and in macrophages, once injected in animals [26]. Although the inflammatory cells are involved in tumor progression and activation through production of pro-tumoral cytokines, control of tumour growth and dissemination by an active immune response cannot be excluded [27]. In the light of new frontiers of cancer immunotherapy, to combat the tumor cells by a combination of immune activation and the induction of an inflammation within the tumor to recruit immune cells seems to be a priority. Although unspecific immune activation in the blood needs to be carefully avoided by any injected nanomaterial, the local immune activation could represent a benefit for cancer treatment [28].

## Conclusion

The present paper reports the safety of CMC hydrogel containing NPs, indicating the possibility of future clinical exploitation of hybrid hydrogels as antitumor and vascular targeting drug delivery systems.

## Supporting Information

S1 Fig Orginal blots of Figs 1, 2, 3, 4. For each figure panel the original blots of the protein of interest and reference marker are reported.
(PDF)
