# Supplementary material for: Monitoring Endothelial and Tissue Responses to Cobalt Ferrite Nanoparticles and Hybrid Hydrogels
Source: PLoS One. 2016 Dec 30;11(12):e0168727. doi: 10.1371/journal.pone.0168727 (PMC5201301; doi:10.1371/journal.pone.0168727)

Supporting information S1

Orginal blots of Figs 1, 2, 3, 4

Figure 1C: Expression of caspase-3 in HUVEC exposed to NPs (0.25 mg/ml). Cells were exposed to the biomaterials for 24 h and then lysed. (1: Ctr; 2: $CoFe_2O_4$; 3. $CoFe_2O_4$-NH_2)

Representative blot of the first version of the paper:

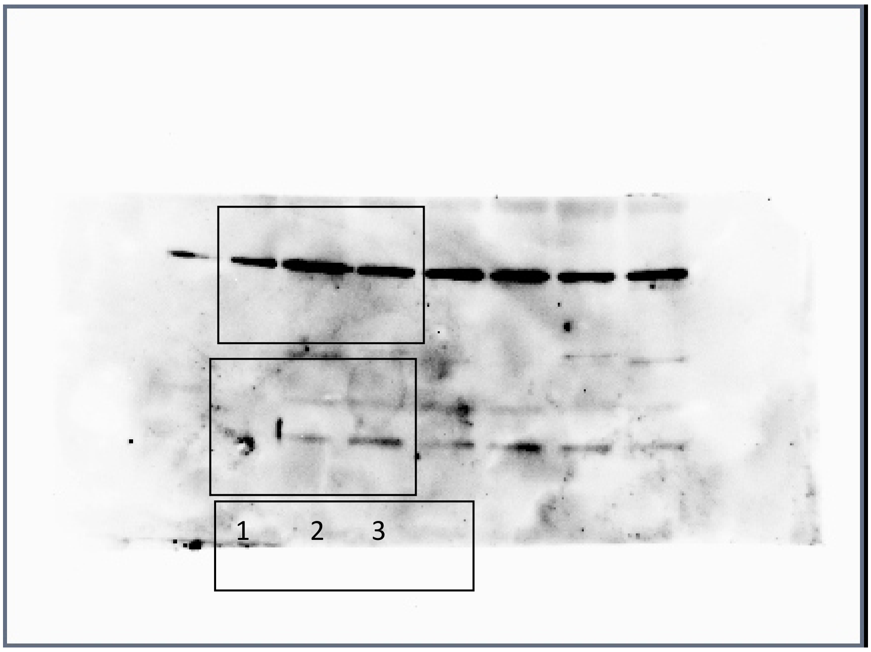

Blank:

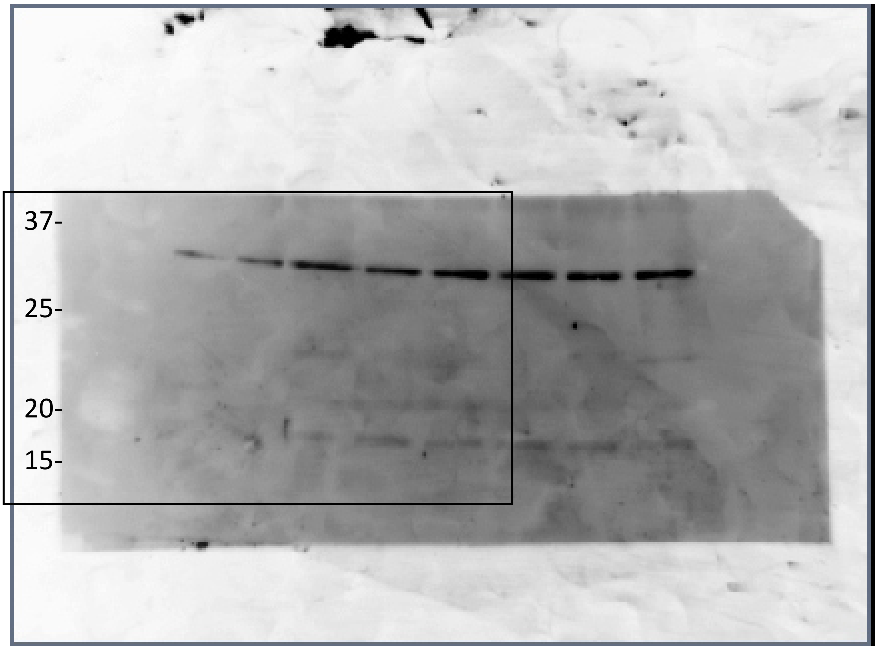

Second representative image:

Cleaved caspase -3

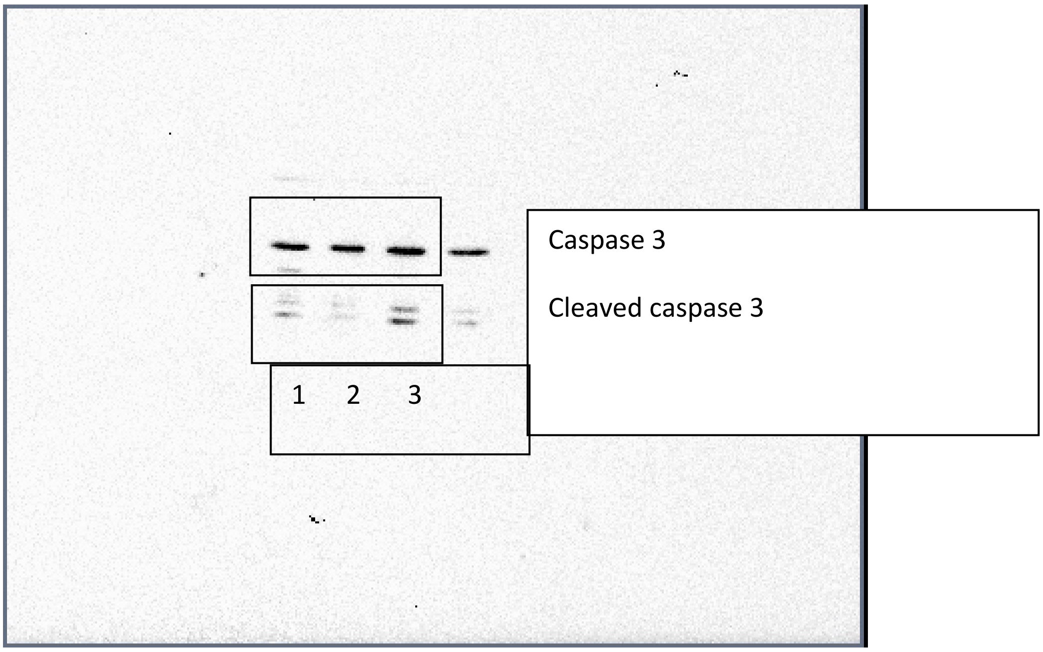

Blank

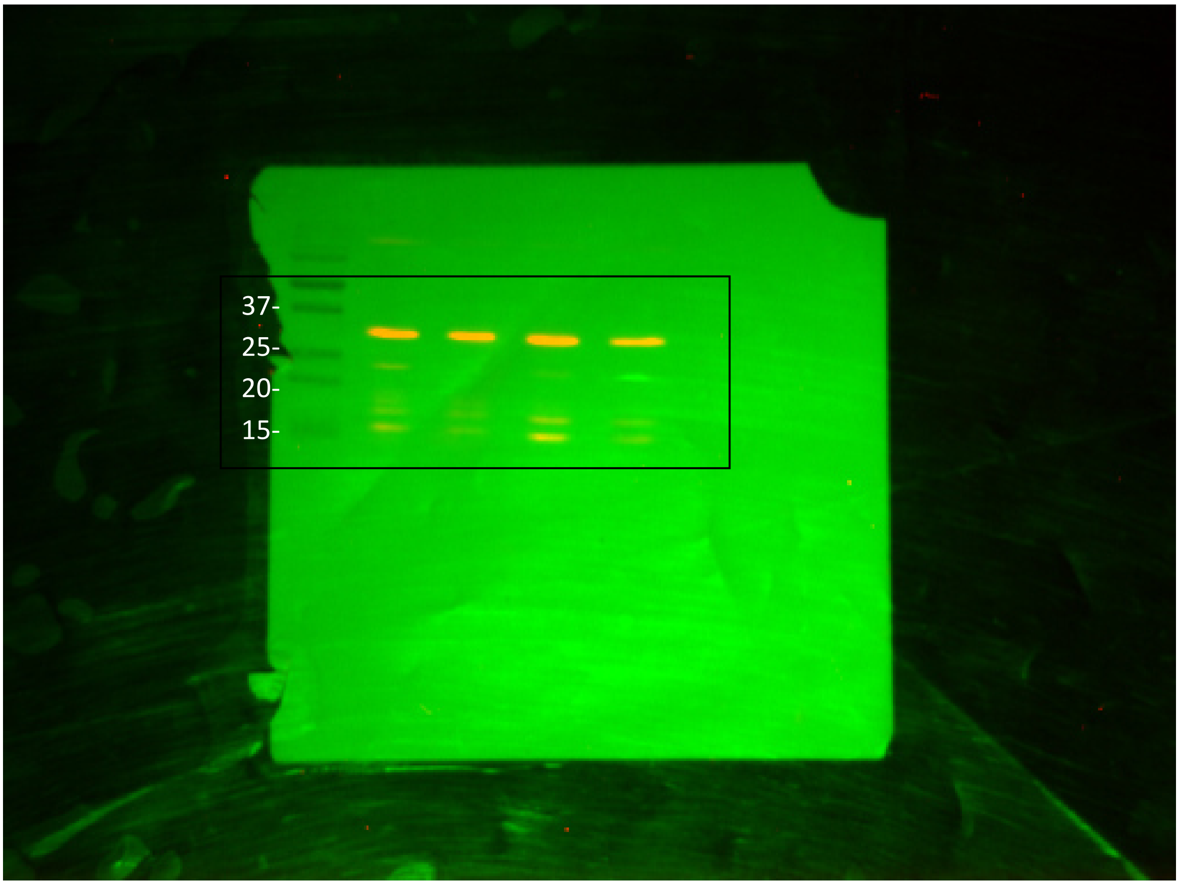

Figure 1E: Expression of p21 in HUVEC exposed to NPs (0.25 mg/ml). Cells were exposed to the biomaterials for 24 h and then lysed. (1: Ctr; 2: $CoFe_2O_4$; 3. $CoFe_2O_4$-NH_2)

P21

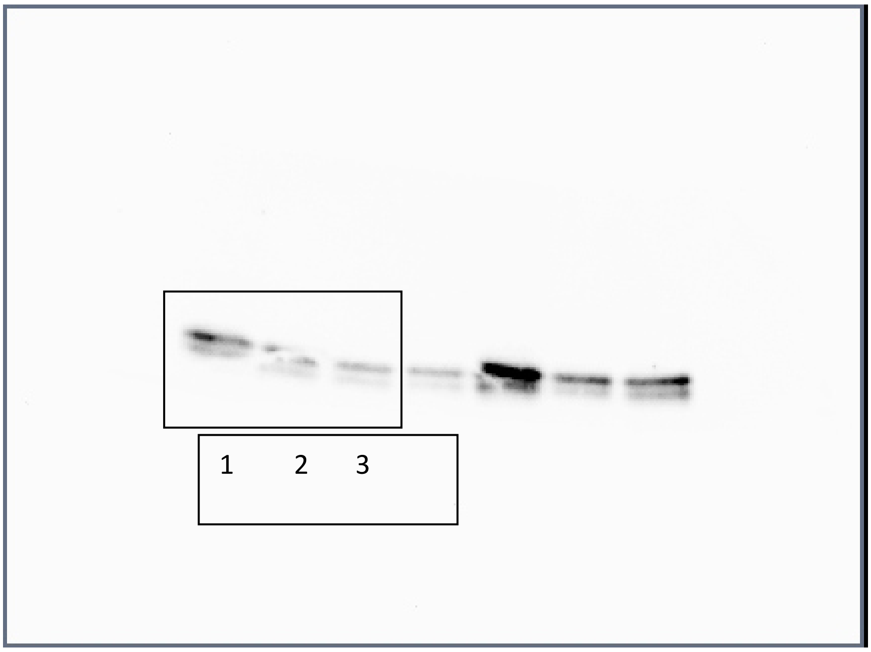

β-Actin:

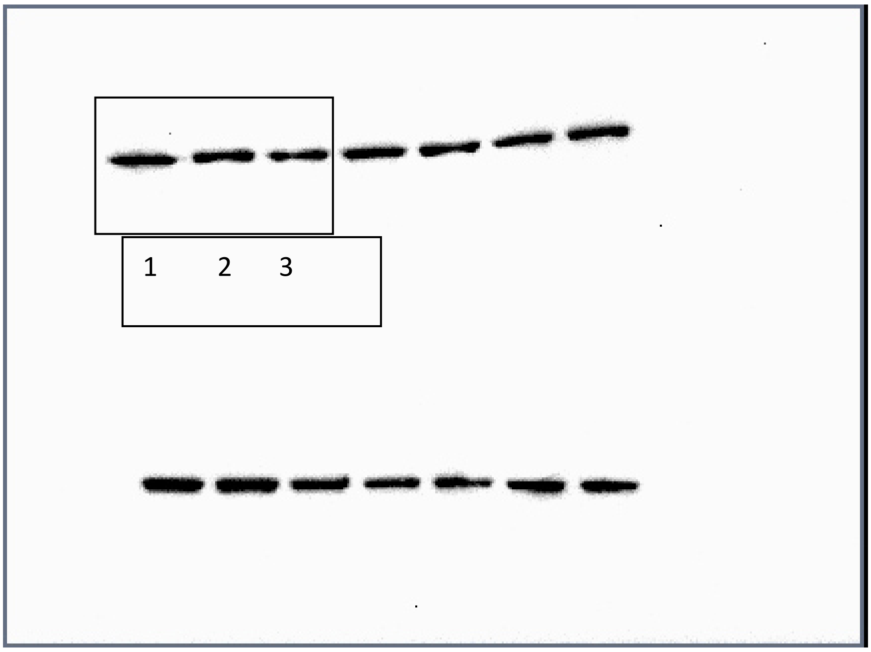

Figure 1F: Expression of p53 in HUVEC exposed to NPs (0.25 mg/ml) . Cells were exposed to the biomaterials for 24 h and then lysed. (1: Ctr; 2: $CoFe_2O_4$; 3. $CoFe_2O_4$-NH_2)

P53

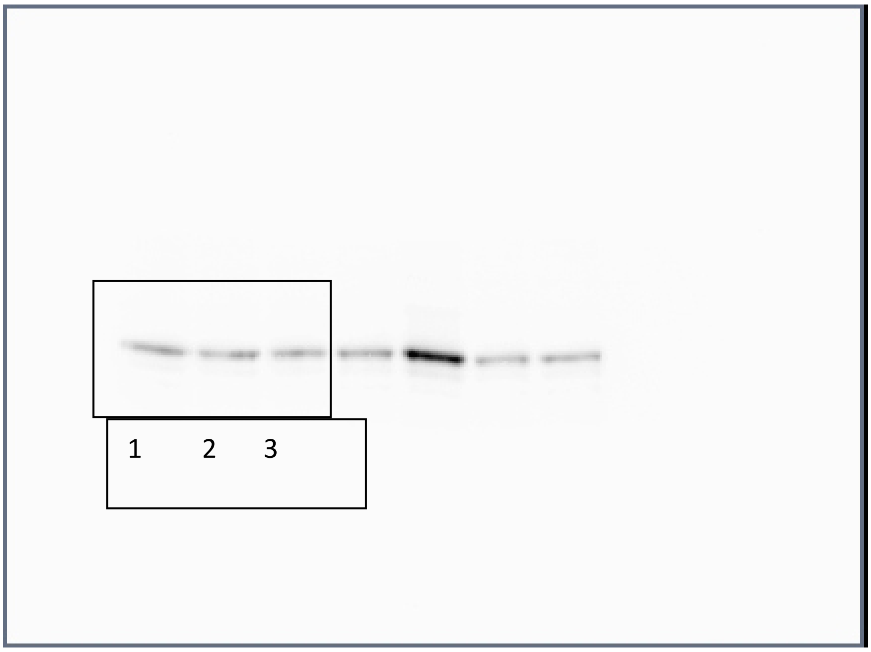

β-Actin:

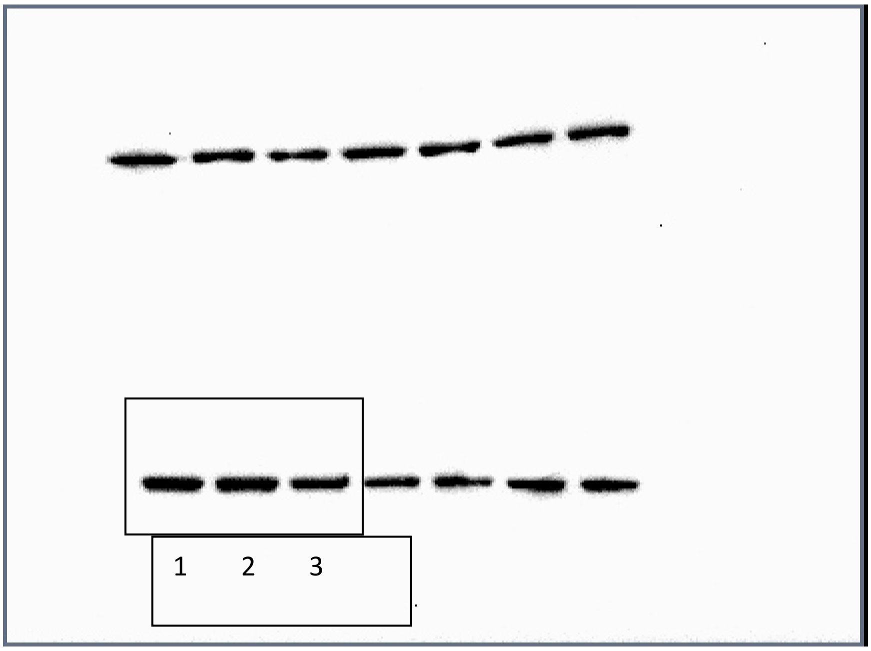

Fig 2D: Expression of COX-2 and iNOS in HUVEC exposed for 24 h to $CoFe_2O_4$ NPs or $CoFe_2O_4$-NH_2 NPs (0.25 mg/ml) measured by western blot. Blots are representative of 3 experiments with overlapping results. (1: Ctr; 2: $CoFe_2O_4$; 3. $CoFe_2O_4$-NH_2)

COX-2:

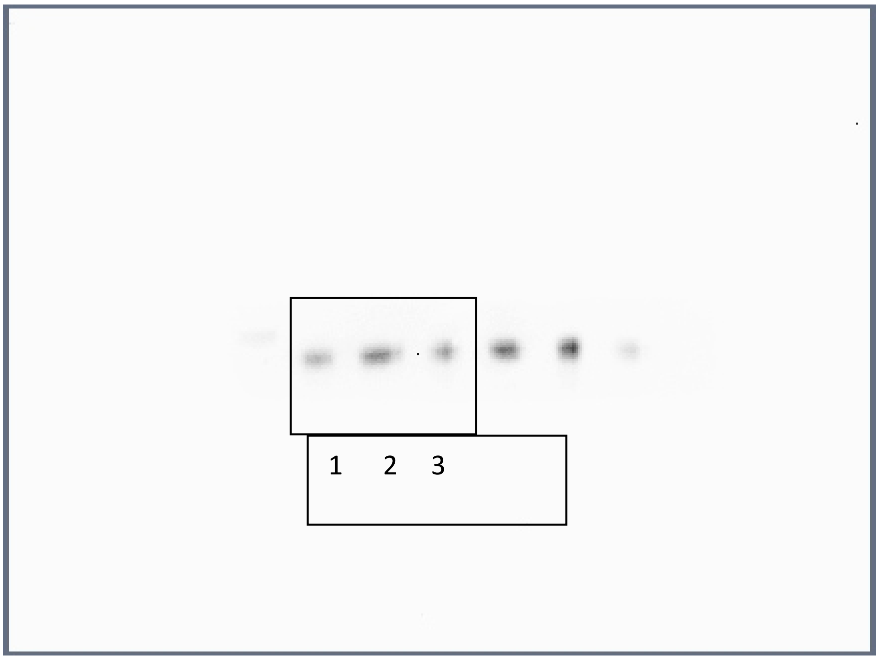

iNOS:

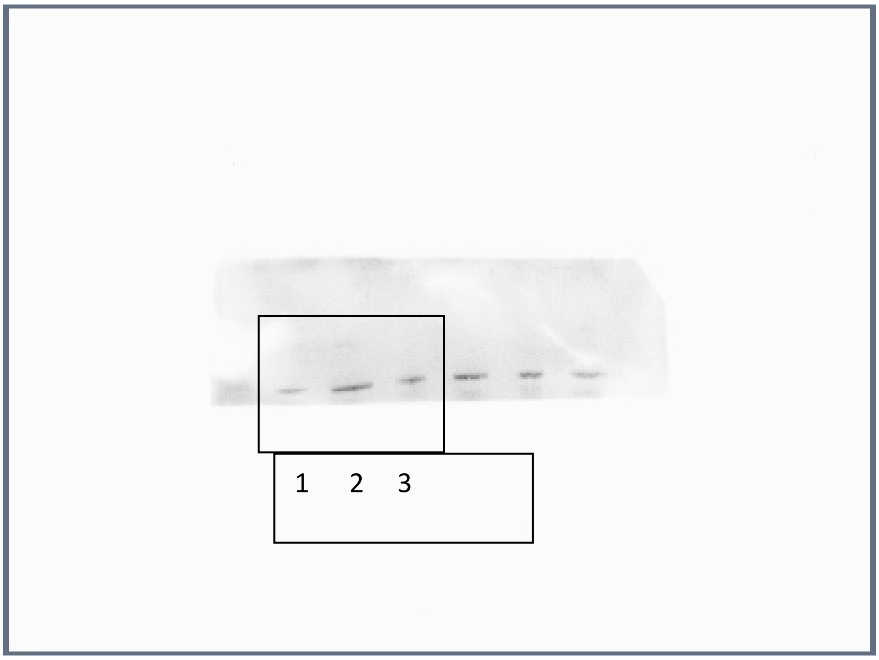

β-Actin:

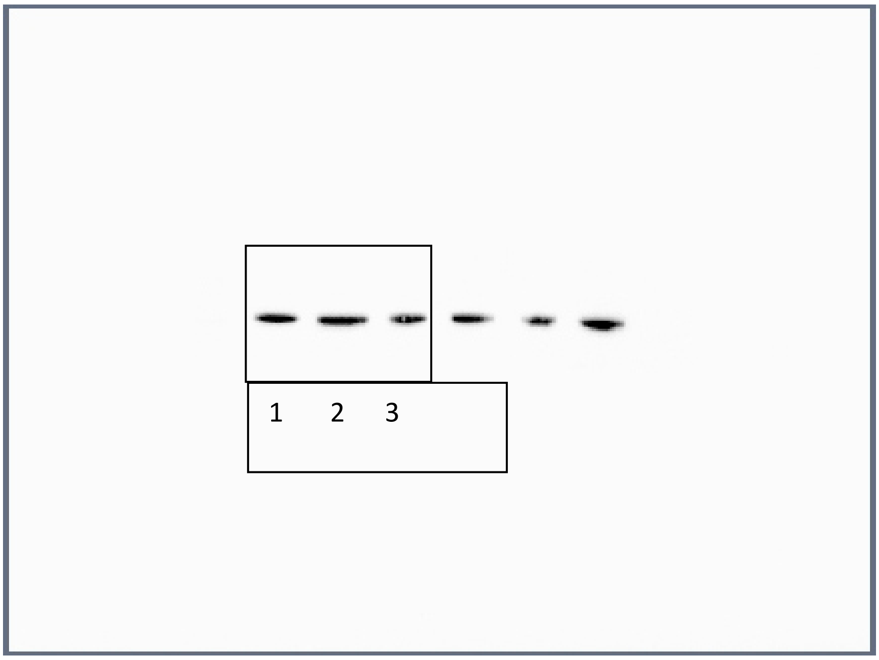

Fig 3C: Evaluation by western blot of the expression of cleaved caspase-3 in HUVEC exposed to CMC (1), $CoFe_2O_4$-CMC (2) or $CoFe_2O_4$-NH_2-CMC (3) (0.5 mg/ml) for 24 h.

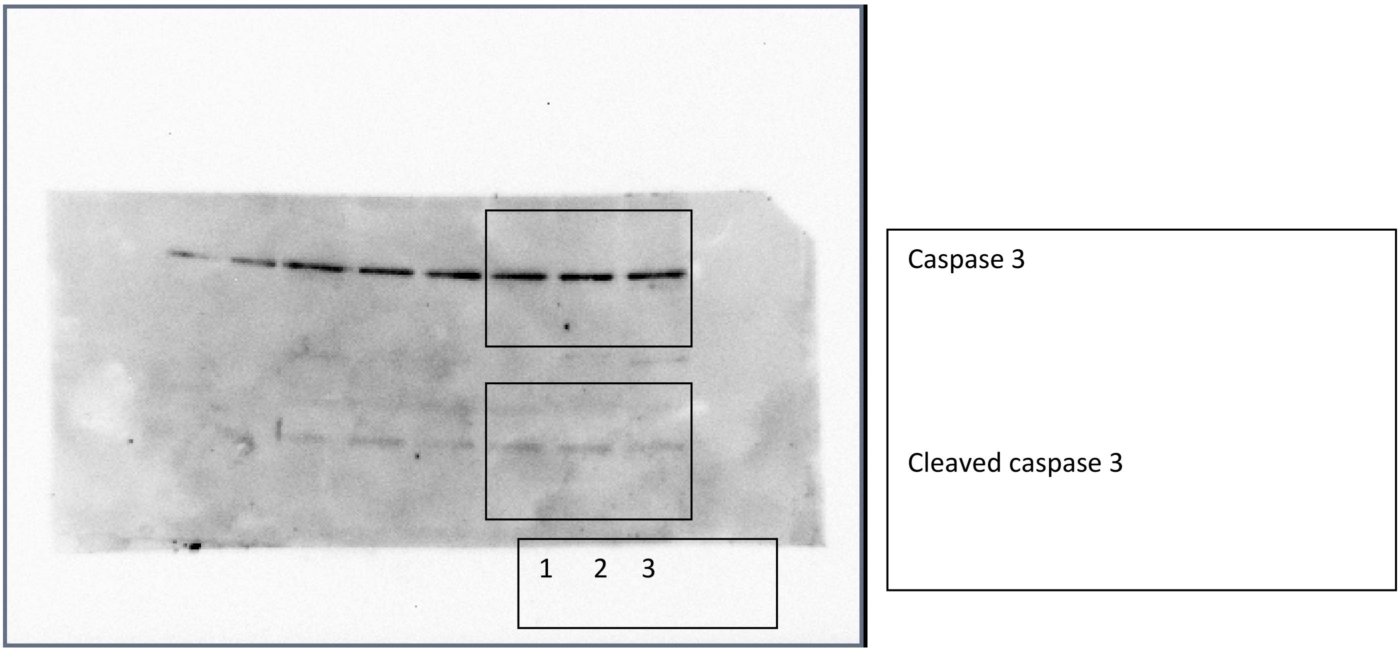

Fig 3E: Evaluation by western blot of the expression of p53 in HUVEC exposed to CMC (1), $CoFe_2O_4$-CMC (2) or $CoFe_2O_4$-NH_2-CMC (3) (0.5 mg/ml) for 24 h.

P53:

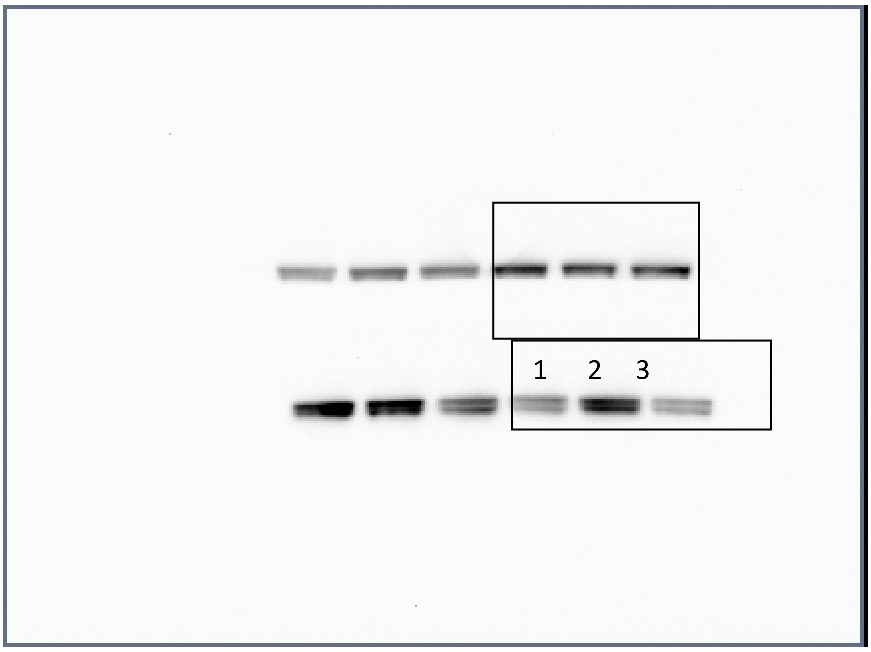

P53 bianco:

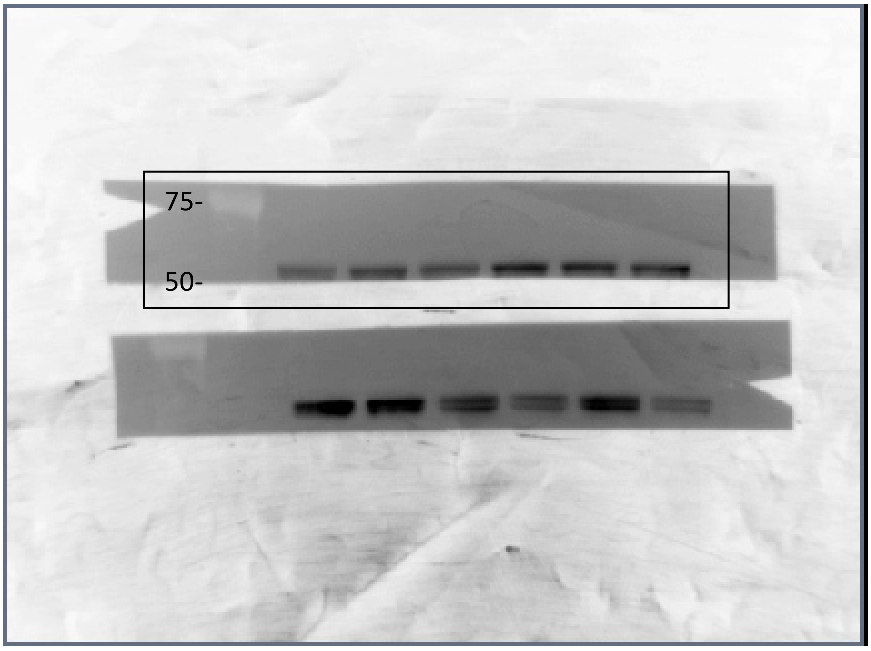

β-Actin:

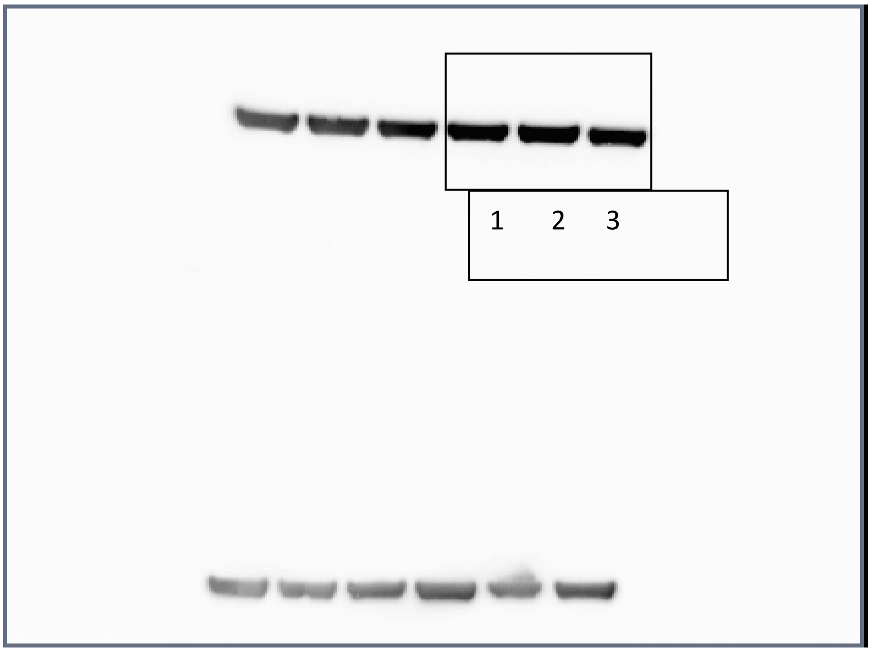

Fig 3F: Evaluation by western blot of the expression of p21 in HUVEC exposed to CMC (1), $CoFe_2O_4$-CMC (2) or $CoFe_2O_4$-NH_2-CMC (3) (0.5 mg/ml) for 24 h.

P21:

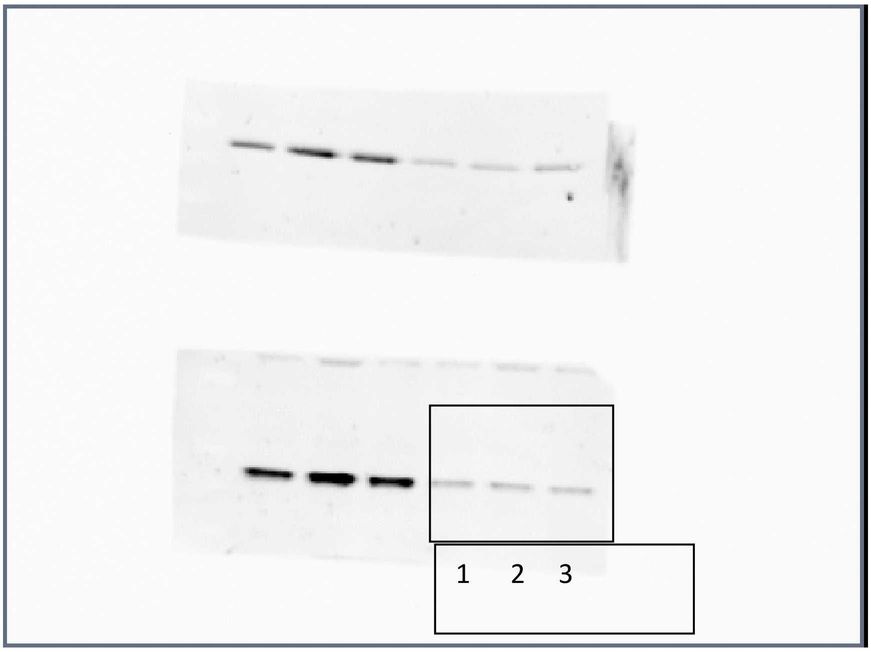

P21 bianco:

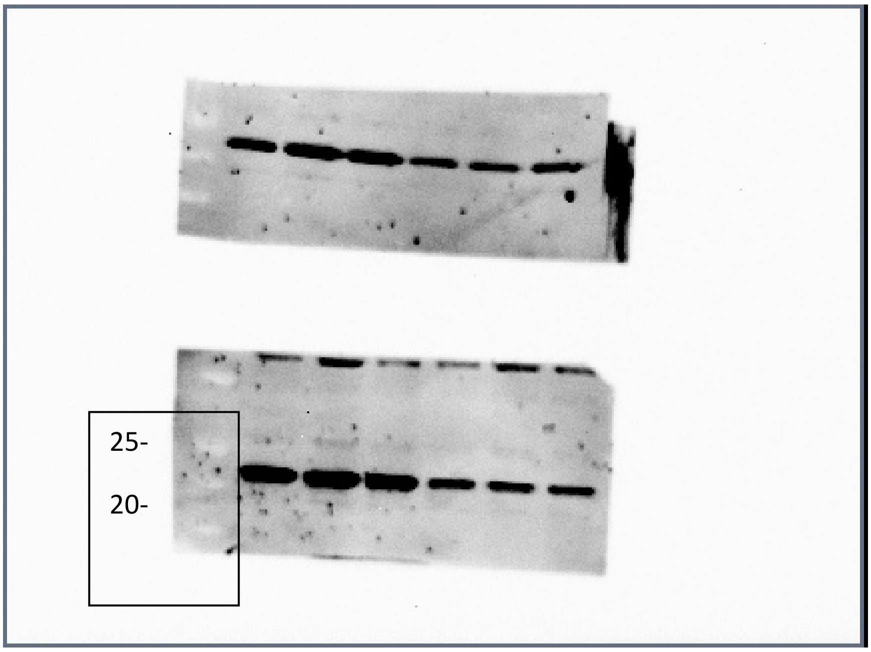

β-Actin:

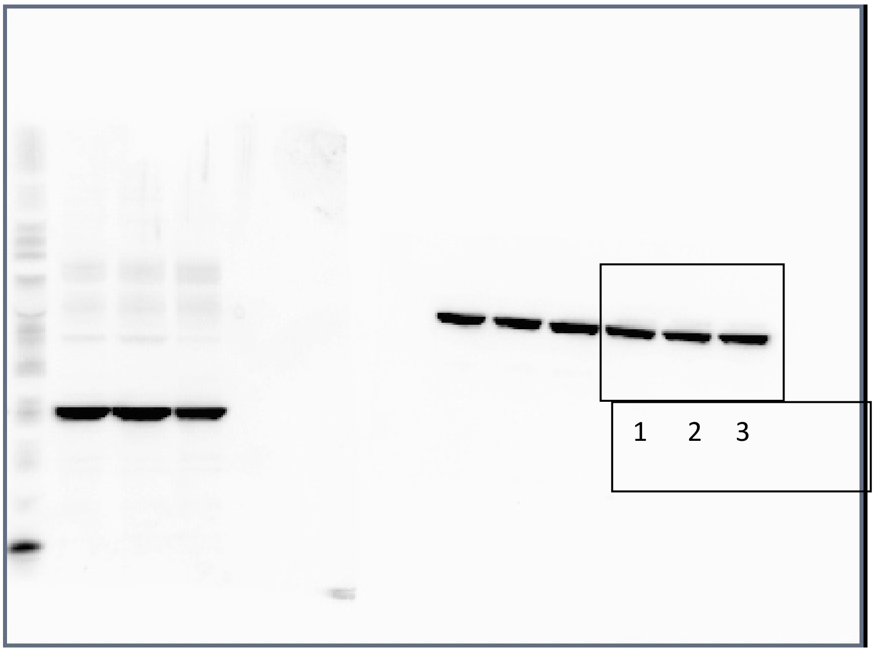

Fig 4B: Expression of COX-2 and iNOS in HUVEC exposed for 24 h to the biomaterials measured by western blot. (1. CMC, 2. $CoFe_2O_4$-CMC, 3. $CoFe_2O_4$-NH_2-CMC, 4. Ctr)

COX-2

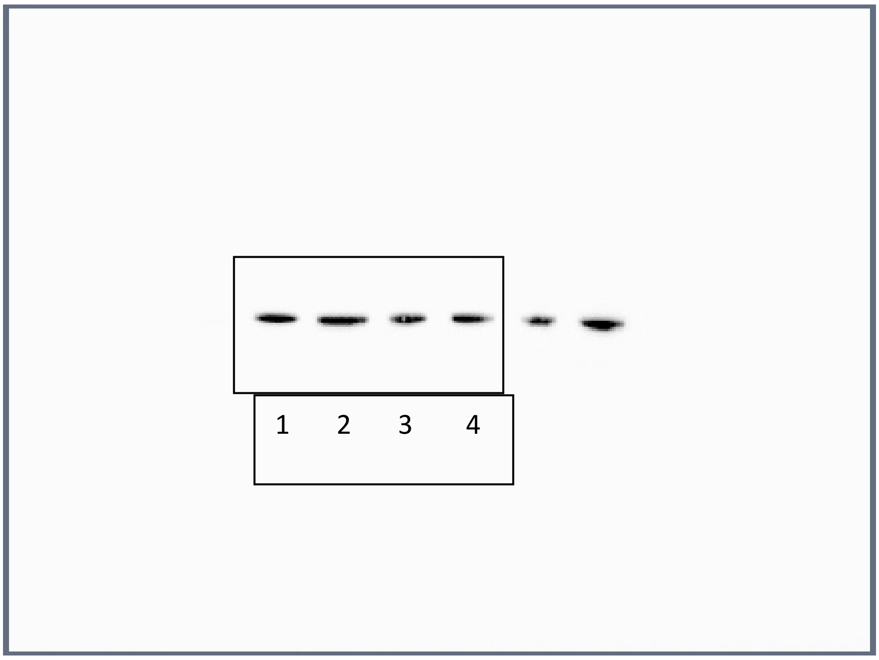

iNOS

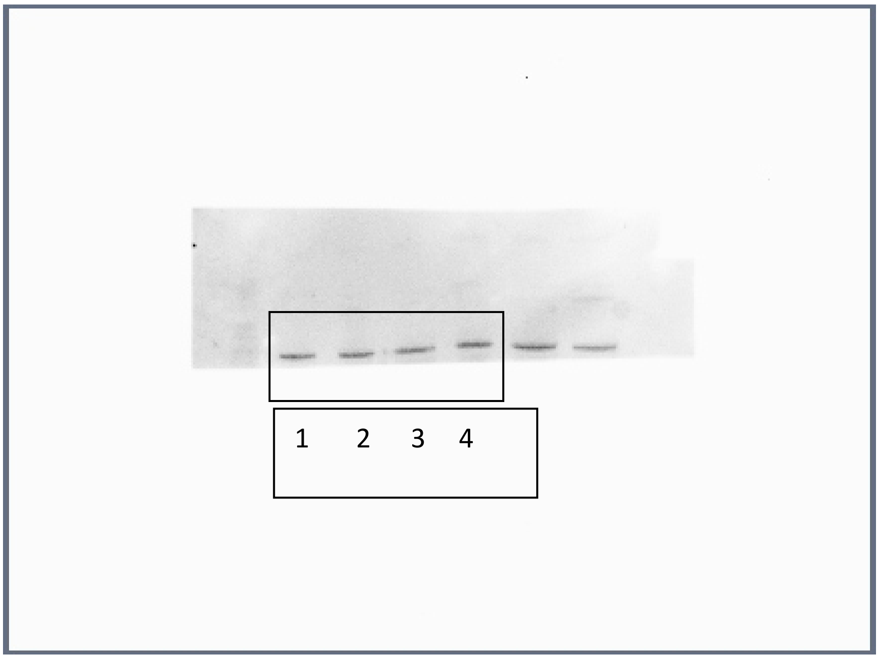

β-Actin:

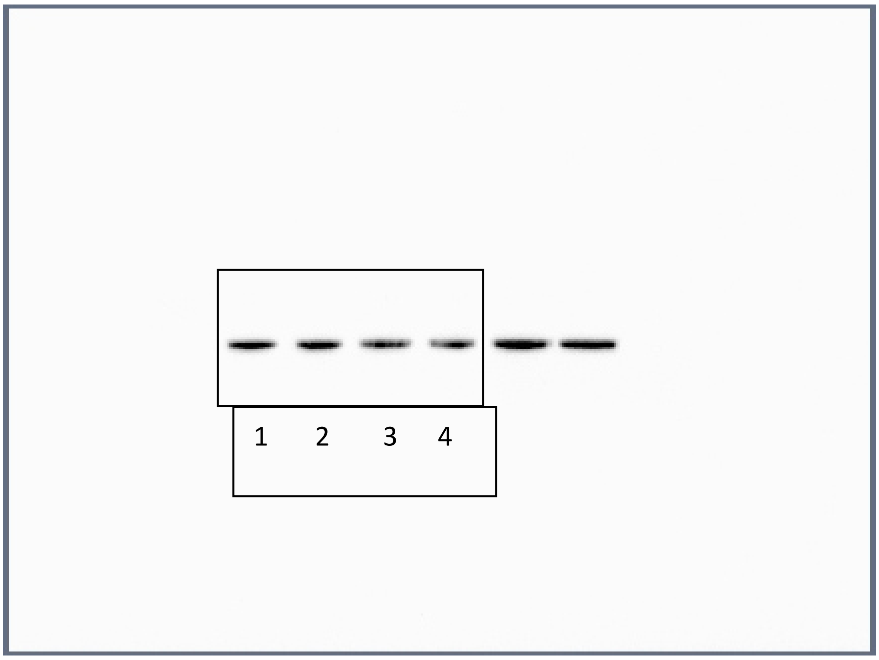

Supplement: S1 Fig — For each figure panel the original blots of the protein of interest and reference marker are reported. (PDF) [file pone.0168727.s001.pdf]
